# Neighborhood Social Support and Social Participation as Predictors of Dating Violence

**DOI:** 10.1177/08862605231155130

**Published:** 2023-02-20

**Authors:** Paul Rodrigues, Martine Hébert, Mathieu Philibert

**Affiliations:** 1 Université du Québec à Montréal, Canada

**Keywords:** dating violence, adolescent, neighborhood, social support, social participation

## Abstract

Many adolescents experience violence in the context of dating and romantic relationships. Neighborhoods can influence dating violence by offering certain resources which can provide social support and opportunities for social participation, but knowledge about these effects is still limited. The purpose of the current study was to (a) assess the association between neighborhood social support, social participation, and dating violence, and (b) explore possible gender difference in these associations. This study was conducted on a subsample of 511 participants living in Montréal from the *Québec Health Survey of High School Students* (QHSHSS 2016–2017). QHSHSS data were used to measure psychological and physical/sexual violence (perpetration and victimization), neighborhood social support, and social participation, as well as individual and family covariates. Several neighborhood-level data from multiple sources were also used as covariates. Logistic regressions were performed to estimate associations between neighborhood social support and social participation, and Dating violence (DV). Analyses were conducted separately for girls and boys to explore possible gender differences. Findings suggest that girls who reported high neighborhood social support had a lower risk of perpetrating psychological DV. High social participation was associated with a lower risk of perpetrating physical/sexual DV for girls, whereas it was associated with a higher risk of perpetrating psychological DV for boys. Preventive strategies to foster social support in neighborhoods, such as mentoring programs, and the development of community organizations to increase the social participation of adolescents could help reduce DV. To address the perpetration of DV by boys, prevention programs in community and sports organizations targeting male peer groups should also be developed to prevent these behaviors.

## Introduction

Dating violence (DV) is a major public health problem among adolescents. Several studies suggest that more than half of adolescents are likely to experience, as a victim or a perpetrator, at least one episode of violence within the context of their intimate and romantic relationships during their lifetime (Taylor & Mumford, 2014; Ybarra et al., 2016). In addition, DV is associated with many consequences, which may be widespread in the population because of this high prevalence. DV is associated with an increased risk of depression and suicidal ideation (Exner-Cortens et al., 2013). It may also influence the onset of at-risk behaviors, such as substance use and risky sexual behaviors (e.g., unprotected sex, a high number of sexual partners) (Shorey et al., 2015). Finally, experiencing DV in adolescence has been identified as a vulnerability factor associated to a higher risk of experiencing intimate partner violence in adulthood (Exner-Cortens et al., 2013). Exner-Cortens et al. (2013) found that adolescent victims and perpetrators in adolescence were twice (for girls, odds ratio [OR] = 1.87; for boys, OR = 2.08) and three times (for girls, OR = 2.79; for boys, OR = 3.56) as likely to be victimized in adulthood, respectively. Identifying risk and protective factors is therefore essential to inform prevention efforts aiming at reducing the prevalence of DV and break the cycle of violence. Given the strong effect of DV experiences in adolescence on DV experiences in adulthood, adolescence appears to be a critical period to implement efficient prevention initiatives.

Empirical studies related to DV mainly focused on exploring individual characteristics (e.g., substance abuse), family characteristics (e.g., exposure to interparental violence), and peer characteristics (e.g., affiliation with deviant peers) as possible risk factors (Hébert et al., 2019; Vagi et al., 2013; Vézina & Hébert, 2007). However, the effects of these determinants remain small and other factors operating at different ecological levels could influence DV (Bronfenbrenner, 1977; Centers for Disease Control and Prevention, 2022; Vagi et al., 2013; Vézina & Hébert, 2007). Characteristics of the neighborhood of residence have not received as much attention as potential determinants of DV, despite multiple studies reporting on associations between such characteristics and violent and delinquent behavior among adolescents (Leventhal et al., 2009).

A few studies suggest that neighborhood characteristics, such as sociodemographic composition (e.g., neighborhood poverty), physical environment (e.g., density of alcohol outlets), and social environment (e.g., collective efficacy) may be associated with DV (Johnson et al., 2015). Among these studies, only a small number focused on the associations between characteristics of the *social* environment and DV. Informed by the social disorganization theory (Sampson et al., 1997), most of them analyzed neighborhoods’ collective efficacy (i.e., the ability of a community to act collectively) and reported mixed results (Johnson et al., 2015). However, neighborhood social environment refers to many concepts, including collective efficacy, social cohesion, social norms, social capital, social support, and social participation (Carpiano, 2006; Diez Roux & Mair, 2010). Thus, knowledge about the role of neighborhood social environment on DV is still limited.

Social environment factors that are potentially influential on DV include neighborhood social support and social participation. Neighborhood social support refers to the supportive resources from neighbors, such as emotional support (Carpiano, 2006; Kawachi et al., 2008). Social participation is defined as community involvement, such as participation in social or leisure activities (Carpiano, 2006; Putnam, 2000). These factors may promote prosocial behaviors in adolescents and adult supervision, which could help monitor and support adolescent (Banyard et al., 2006; Browning & Soller, 2014; Quane & Rankin, 2006). They may also facilitate disclosure and help-seeking for victims (Banyard et al., 2006; Browning, 2002; Wright et al., 2015). However, little is known about the effect of neighborhood social support and social participation on DV specifically.

To date, empirical studies of associations between social support and DV focused on social support from family and friends and found relatively small effects (Hébert et al., 2019; Richards & Branch, 2012; Richards et al., 2014). Only one study assessed the effect of neighborhood-level social support on DV (Banyard et al., 2006) and reported nonsignificant associations with physical and sexual DV perpetration (psychological DV was not evaluated) after accounting for individual factors. To our knowledge, the relationship between *neighborhood* social support and DV victimization has not yet been analyzed. Still, a protective effect of neighborhood social support on psychological intimate partner violence has been reported (Kirst et al., 2015).

We are unaware of any study examining the association between social participation and DV among adolescents. However, two studies explored the association in adult populations. One study found no significant association between social participation and intimate partner violence perpetrated by men on women (Voith & Brondino, 2017). The other study reported no significant association between social participation and psychological partner violence victimization but reported a positive association between social participation and physical violence victimization, suggesting potential reverse causality, as victims may seek more support, such as seeking to join a group to cope with social isolation (Kirst et al., 2015). Further exploration of these relationships among adolescents would contribute to the body of knowledge about the determinants of DV and identify new targets for intervention.

### Neighborhood Social Support, Social Participation, and DV

Neighborhood social support and social participation are likely to affect individuals’ health and behaviors (Carpiano, 2006) and may influence DV through several mechanisms. Social support can take many forms (e.g., emotional, instrumental) that individuals can mobilize to cope with specific problems (Carpiano, 2006; Kawachi et al., 2008). Individuals may obtain such resources through their social networks. To date, studies mainly focused on the association between social support from peers or family and DV (Hébert et al., 2019; Richards & Branch, 2012; Richards et al., 2014). However, the social support network is broader, and the size and diversity of social support networks could influence some processes related to violence in romantic relationships (e.g., disclosure, help-seeking) (Sylaska & Edwards, 2014; Nolet et al., 2021). It is therefore essential to consider social support from sources other than peers and family, such as social support from neighborhood networks. Neighborhood social support would have many positive effects (Carpiano, 2006) and may reduce the risk of experiencing DV. A high social support in neighborhoods could, for instance, contribute to adolescents’ supervision, facilitate disclosure and help-seeking for victims, and protect individuals from abusive relationships (Browning, 2002; Wright et al., 2015).

Social participation refers to residents’ involvement in formally organized activities (e.g., sports clubs, community organizations, religious organizations) (Carpiano, 2006; Putnam, 2000). Adolescent social participation may allow for greater adult supervision, promote the development of prosocial skills, and provide opportunities to develop social relationships (Browning & Soller, 2014; Quane & Rankin, 2006). Involvement in structured activities may also help reduce adolescents’ exposure to negative aspects of their neighborhood, such as violence (Gardner & Brooks-Gunn, 2009). Social participation could therefore have many benefits for adolescents by contributing, for example, to higher self-esteem, a decrease in delinquent behavior, and a reduction in mental health problems (Bohnert et al., 2008; Farb & Matjasko, 2012), all of which have been highlighted as potential protective factors of DV (Vagi et al., 2013; Vézina & Hébert, 2007).

### Effect Modification by Gender

In studying the relationship between neighborhood characteristics and DV, gender differences are often observed, suggesting a modifying effect (Chang et al., 2015; Jain et al., 2010; Schnurr & Lohman, 2013). One possible explanation for this gender sensitivity is gender-based parental attitudes. Parents could exert greater supervision and impose more restrictive rules about going out to girls compared to boys (Axinn et al., 2011). As a result, boys may explore their neighborhoods more and thus be more exposed to their neighborhoods’ positive and negative aspects (Kupersmidt et al., 1995; Leventhal et al., 2009). Gender modification of the effect of neighborhood social support and social participation may also be due to the differences in the nature and functions of the social network and the characteristics of peer groups. Gender norms could affect social practices, which could influence social network density and social support expectations. For example, girls may seek greater closeness and intimacy with their peers, while boys may prefer a larger and less intimate social network (Kuttler et al., 1999; Richards & Branch, 2012). In addition, gender may also alter the type of social participation, and these differences may influence adolescents’ behavior. Activities vary by gender and involve the use of different environments (e.g., sports clubs, social organizations). Peer groups within these environments would tend to be different in nature and would create different norms and values, reflecting the male and female peer groups’ cultures (Barber et al., 2005; Brown, 1990; Eccles et al., 2003). Therefore, the effect of social participation on DV could vary by gender following different peer group affiliations of girls and boys. For example, while sports participation may protect girls from DV due to the nonacceptance of the traditional gender norms in some female athletic groups (Milner & Baker, 2015), it may be associated with increased perpetration of DV by boys due to sexist social norms in male athletic groups (De Keseredy & Kelly, 1995).

### Objectives

In the research on the determinants of DV, studies on neighborhood social support are few, and the effect of social participation has been overlooked. The first goal of this study was to analyze the association between neighborhood social support and social participation and DV (victimization and perpetration). The second objective was to assess the modifying effect of gender on these associations.

The hypothesis was that a higher neighborhood social support and social participation would be associated with a lower risk of DV perpetration and victimization. Related to previous literature, it was also hypothesized that these effects would vary by gender. We thus expected that the effect of neighborhood social support would be stronger for girls and that high social participation would be associated with a lower risk of DV for girls and a higher risk of DV for boys.

## Methods

### Participants

Data were drawn from the Québec Health Survey of High School Students (QHSHSS, 2016–2017), a province-wide, cross-sectional survey of the health and well-being of high school students. In this study, we restricted the analyses to the island of Montréal and excluded respondents without a valid postal code as the postal code was required to assign neighborhood-level variables to participants (see *covariates* below). The QHSHSS used a three-level stratified cluster sampling design. Within strata of grade level, schools were randomly sampled with a probability of selection proportional to the number of students. Then, for each school selected, classes were randomly and independently selected for each grade. The response rate among participants was 91%.

Due to the large number of topics covered by the QHSHSS, two questionnaire versions were randomly assigned in equal proportions to students in each class. To reduce sampling bias, participants were assigned a weight for each questionnaire version. This study uses the weighted sample from version 2 of the questionnaire (*n* = 1,361), which included questions about the social support in the neighborhood and social participation.

### Dependent Variable

Four variables were used to assess DV: psychological DV victimization, psychological DV perpetration, physical/sexual DV victimization, and physical/sexual DV perpetration. Similar questions were used for victimization (e.g., “he/she . . . me”) and perpetration (“I . . . him/her”). Two items were used to measure psychological DV victimization (i.e., “He/she controlled my outings, my email conversations or cell phone; he/she prevented me from seeing my friends”/“He/she viciously criticized my physical appearance; he/she insulted me in front of people; he/she put me down”). Two other items measured psychological DV perpetration (i.e., “I criticized him/her viciously about his/her physical appearance; I insulted him/her in front of people; I put him/her down.”/“I criticized him/her viciously about his/her physical appearance; I insulted him/her in front of people; I put him/her down”). For these four items, participants were asked to indicate how often they had experienced each situation in the past 12 months using a 4-point Likert scale: “never,” “once,” “twice,” and “three or more times.” Dichotomous variables for victimization and perpetration were developed separately by distinguishing participants who reported never psychological DV perpetration/victimization from those who reported at least one of these situations.

Four items from an adapted version of the Conflict Tactics Scale (Straus et al., 1996) were used to measure physical DV victimization (e.g., “He/she used his/her fists or feet, an object or a weapon to hurt me”) and perpetration (e.g., “I used my fists or feet, an object or a weapon to hurt him/her”). For sexual DV, two items were used for victimization (i.e., “He/she forced me to kiss or caress him/her when I didn’t want to” / “He/she forced me to have sexual contact or sexual intercourse when I didn’t want to”) and two other questions were used for perpetration (i.e., “I forced him/her to kiss or caress me when he/she didn’t want to” / “I forced him/her to have sexual contact or sexual intercourse with me when he/she didn’t want to”). Physical and sexual DV were assessed jointly because the number of participants who reported perpetrating sexual violence was relatively low. Similar to the psychological measures of DV, two dichotomous variables (victimization and perpetration) were developed to differentiate participants who had experienced at least one physical/sexual DV event in the past 12 months from those who reported never having experienced one.

### Independent Variables

#### Neighborhood social support and social participation

Neighborhood social support was measured using six questions inspired by the *California Healthy Kids Survey* (Austin et al., 2013). For each question, participants were asked to think about situations that might occur outside their school or home, such as in their neighborhood. The statements assessed the adolescents’ perception of their relationships with adults outside the home and school (e.g., “Outside of my home and school, there is an adult who really cares about me”). Four response options were available, ranging from 1 (“*Not at all*”) to 4 (“*Very much*”). The scores on the six variables were averaged and dichotomized: low social support (average score below 3) and high social support (average score above 3). Internal consistency was excellent (α = .96).

Social participation was assessed using three questions describing the respondent’s perception of their involvement in community life and their ability to participate in activities (e.g., “Outside of my home and school, I am part of clubs, sports teams, church/temple or other group activities”). The response options ranged from 1 (“*Not at all*”) to 4 (“*Very much*”). Average scores of the three statements were dichotomized to distinguish participants with low social participation (average score below 3) from those with high social participation (average score above 3). Internal consistency was acceptable (α = .72).

#### Covariates

Adjustment variables were selected using a Directed Acyclic Graph (DAG). DAGs are graphical representations of causal relationships between exposures and a given outcome (Pearl, 1995; Schisterman et al., 2009). They are powerful tools that can be used to identify confounders and reduce the risk of overadjustment (Schisterman et al., 2009). The DAG led to identifying potential confounders at both individual and neighborhood levels.

Five variables were used as covariates at the individual level: gender (girl or boy), high school grade level (Grade 7 or 8, 9, 10, and 11), the highest level of parental education (high school or less, college or professional training, university), family structure (two parents, blended family or shared custody, living with one parent or other family structure), and parental country of birth (two parents born in Canada, at least one parent born outside Canada). Several neighborhood-level factors were also identified as potential confounders, describing sociodemographic, physical, and social characteristics associated with DV (Rodrigues et al., 2022): socioeconomic status, single parenthood, residential instability, ethnocultural diversity, greenness, walkability, criminality, density of green spaces, and density of alcohol outlets (on-premises and off-premises). These neighborhood-level variables were operationalized using buffers around the participants’ place of residence (i.e., postal code of participants). Since the effect of these factors is sensitive to the spatial scale of analysis, four distances were used to obtain these buffers: 250, 500, 750, and 1,000 m. Prior analyses were conducted to identify the most appropriate scale for each factor and outcome separately using the models’ fit. Details of the operationalization of neighborhoods’ characteristics are available elsewhere (Rodrigues et al., 2022).

### Statistical Analyses

Among the 1,361 QHSHSS (version 2) participants with a valid postal code on the island of Montréal, 519 adolescents reported being involved in at least one romantic relationship in the past 12 months (38.1% of participants) and were selected for this study. Participants with at least one missing data for measures of DV (*n* = 2), neighborhood social support (*n* = 6), and social participation (*n* = 2) were excluded (1.5% of participants reported a romantic relationship). Missing data for individual-level covariates were handled by multiple imputation methods (10 iterations). The final sample consisted of 511 adolescents.

Neighborhood data were assigned to participants using postal codes. Associations between neighborhood social support and social participation and the four DV measures were estimated in logistic regression models stratified by gender. All models were adjusted by the individual- and neighborhood-level covariates and used the sampling weight. Statistical analyses were performed in SAS Enterprise Guide 8.5 (Sas Institute, 2011).

## Results

### Descriptive Analyses

Table 1 describes the distribution of participants according to sociodemographic characteristics, neighborhood social support and social participation, and neighborhood-level characteristics. Most adolescents lived in two-parent families (62.71%), and their parents’ highest level of education was university (58.84%). The perpetration of psychological DV was reported by 19.80% of adolescents, and the physical/sexual form concerned 14.84% of the participants. For victimization, 26.49% of youth reported having experienced psychological DV, while 19.74% of teenagers reported having experienced physical/sexual DV. These proportions are significantly higher for girls than for boys. Finally, a majority of adolescents (63.75%) reported a high social participation, with girls reporting a significantly higher prevalence than boys (67.84% vs. 59.51%; χ^2^ = 3.835; *p* = .050).

**Table 1.** Sample Description.

	Girls (*n* = 269)	Boys (*n* = 242)	
Individual-Level Variables			
	%	%	Chi-Square^a^
Psychological DV perpetration			6.632*
At least once	24.04	14.99	
Never	75.96	85.01	
Physical/sexual DV perpetration			7.964**
At least once	19.16	10.03	
Never	80.84	89.97	
Psychological DV victimization			4.466*
At least once	30.54	22.28	
Never	69.46	77.72	
Physical/sexual DV victimization			8.635**
At least once	24.82	14.47	
Never	75.18	85.53	
Grade level			3.528
Grade 7 or 8	27.61	32.78	
Grade 9	18.89	20.03	
Grade 10	21.80	22.57	
Grade 11	31.70	24.61	
Parental country of birth			1.635
Two parents born in Canada	36.11	39.56	
At least one parent born outside Canada	59.67	51.48	
Family structure			1.495
Two parents	61.10	64.37	
Blended family or shared custody	19.77	15.64	
Living with one parent or other family structure	19.12	19.99	
Highest level of parental education			5.879****
High school or less	16.72	10.28	
College or professional training	22.24	18.38	
University	55.26	62.55	
Neighborhood social support			0.417
High	52.48	49.62	
Low	47.52	50.38	
Social participation			3.835*
High	32.16	40.49	
Low	67.84	59.51	
Neighborhood-Level Variables			
	Mean (SD)	Mean (SD)	T^2^
250 m			
Sociodemographic characteristics			
Median income	57100.88 (23034.80)	56780.45 (21506.75)	0.08
Single parenthood	20.69 (7.52)	20.27 (7.97)	–0.79
Residential instability	39.70 (12.60)	38.94 (13.61)	–0.78
Ethnocultural diversity	1.28 (0.37)	1.27 (0.39)	–0.21
Population density	10.02 (5.90)	10.33 (6.90)	0.23
Risk factors			
Density of off-premises alcohol outlets	0.78 (1.12)	0.77 (1.23)	–0.28
Density of bars	0.37 (1.02)	0.29 (0.91)	–0.67
Crime rate	1.58 (1.18)	1.42 (1.27)	–1.20
Protective factors			
Walkability	–0.17 (2.54)	–0.48 (2.65)	–1.86****
NDVI	0.49 (0.10)	0.50 (0.11)	0.98
Density of green spaces	0.86 (0.92)	0.85 (0.84)	0.17
Density of community organizations	0.43 (0.69)	0.36 (0.64)	–1.34
500 m			
Sociodemographic characteristics			
Median income	56427.33 (20695.65)	56003.61 (19066.91)	0.03
Single parenthood	20.77 (6.52)	20.16 (6.51)	–1.13
Residential instability	39.97 (11.37)	39.66 (12.35)	–0.55
Ethnocultural diversity	1.28 (0.36)	1.27 (0.38)	–0.40
Population density	8.85 (4.34)	8.80 (4.80)	–0.40
Risk factors			
Density of off-premises alcohol outlets	3.52 (3.97)	3.61 (4.62)	–0.36
Density of bars	1.52 (2.78)	1.59 (3.08)	–0.48
Crime rate	1.23 (0.64)	1.61 (5.90)	0.75
Protective factors			
Walkability	–0.19 (2.64)	–0.51 (2.74)	–1.82****
NDVI	0.49 (0.10)	0.50 (0.10)	0.79
Density of green spaces	2.98 (2.19)	2.64 (1.81)	–1.65****
Density of community organizations	1.23 (1.23)	1.14 (1.29)	–1.01
750 m			
Sociodemographic characteristics			
Median income	55627.28 (18599.37)	55487.68 (18152.49)	0.16
Single parenthood	20.93 (5.78)	20.33 (5.90)	–1.18
Residential instability	40.36 (10.46)	39.9 (11.63)	–0.70
Ethnocultural diversity	1.29 (0.35)	1.28 (0.37)	–0.45
Population density	8.37 (3.71)	8.07 (4.09)	–1.02
Risk factors			
Density of off-premises alcohol outlets	7.90 (7.85)	7.62 (8.12)	–0.91
Density of bars	3.85 (6.44)	3.54 (5.79)	1.07
Crime rate	1.20 (0.56)	1.18 (0.62)	–0.69
Protective factors			
Walkability	–0.21 (2.64)	–0.55 (2.75)	–1.74****
NDVI	0.49 (0.09)	0.49 (0.10)	0.66
Density of green spaces	5.91 (3.91)	5.35 (3.20)	–1.67****
Density of community organizations	2.24 (1.95)	2.15 (2.03)	–1.00
1,000 m			
Sociodemographic characteristics			
Median income	55095.52 (16571.17)	55013.66 (16799.17)	0.12
Single parenthood	21.01 (5.38)	20.55 (5.41)	–0.84
Residential instability	40.89 (9.95)	40.11 (10.84)	–1.04
Ethnocultural diversity	1.30 (0.33)	1.29 (0.35)	–0.35
Population density	7.89 (3.34)	7.52 (3.62)	–1.25
Risk factors			
Density of off-premises alcohol outlets	13.74 (13.25)	12.95 (12.72)	–1.10
Density of bars	7.31 (13.03)	6.63 (11.36)	–1.15
Crime rate	1.19 (0.53)	1.17 (0.54)	–0.51
Protective factors			
Walkability	–0.23 (2.62)	–0.62 (2.75)	–1.97*
NDVI	0.49 (0.09)	0.49 (0.09)	0.52
Density of green spaces	9.82 (6.44)	8.74 (5.10)	–2.31*
Density of community organizations	3.65 (3.00)	3.59 (2.91)	–0.86

*Note*. The sum of some proportions does not equal 100 due to missing data. DV = Dating violence.

a Chi-square test for comparison of proportions in girls and boys.

b *T*-test for comparison of girls’ and boys’ averages.

* *p* < .05. **p < .01; ***p < .001. ****p < .10.

### Effect of Neighborhood Social Support and Social Participation on DV in Girls

Table 2 shows the results of the logistic regressions analyzing the association between neighborhood social support and social participation, and DV among girls. Girls reporting high neighborhood social support were less likely to perpetrate psychological DV than those who reported low neighborhood social support (OR = 0.61; 95% CI [0.42–0.90]; *p* = .012). No significant associations were observed between neighborhood social support and physical/sexual DV perpetration nor with the two measures of DV victimization.

**Table 2.** Associations Between Neighborhood Social Support, Social Participation, and DV.

	Girls			
	Perpetration		Victimization	
	Psychological	Physical/sexual	Psychological	Physical/sexual
	OR (95% CI)	OR (95% CI)	OR (95% CI)	OR (95% CI)
Social support				
Low	Ref.	Ref.	Ref.	Ref.
High	0.61 (0.41–0.89)*	1.04 (0.71**–**1.51)	0.82 (0.61**–**1.09)	1.00 (0.70**–**1.43)
Social participation				
Low	Ref.	Ref.	Ref.	Ref.
High	0.93 (0.64**–**1.36)	0.60 (0.39–0.93)*	1.09 (0.78**–**1.54)	1.05 (0.69**–**1.62)
	Boys			
	Perpetration		Victimization	
	Psychological	Physical/sexual	Psychological	Physical/sexual
	OR (95% CI)	OR (95% CI)	OR (95% CI)	OR (95% CI)
Social support				
Low	Ref.	Ref.	Ref.	Ref.
High	0.93 (0.59**–**1.49)	0.71 (0.42**–**1.20)	0.93 (0.65**–**1.33)	0.89 (0.54**–**1.47)
Social participation				
Low	Ref.	Ref.	Ref.	Ref.
High	1.69 (1.05–2.73)*	1.10 (0.67**–**1.79)	1.30 (0.88**–**1.92)	0.69 (0.41**–**1.14)

*Note*. All models were adjusted for the individual- and neighborhood-level covariates.

DV = Dating violence.

* *p* < .05.

High social participation was associated with a lower likelihood of perpetrating physical/sexual DV among girls (OR = 0.63; 95% CI [0.44**–**0.91]; *p* = .013). There were no significant associations between social participation and other forms of DV.

### Effect of Neighborhood Social Support and Social Participation on DV in Boys

The estimated associations between neighborhood social support and social participation, and DV among boys are summarized in Table 2. Boys reporting high social participation had a greater risk of perpetrating psychological DV than those reporting low social participation (OR = 1.69; 95% CI [1.05**–**2.73]; *p* = .032). No significant associations were found between social support and other forms of DV nor between neighborhood social support and all forms of DV.

## Discussion

DV is a significant public health problem, and our data are consistent with studies suggesting a high prevalence of DV (Taylor & Mumford, 2014; Ybarra et al., 2016). Psychological DV was the most frequent form of DV, with 19.6% of the adolescents reporting having been a victim and 26.5% reporting having perpetrated it. This high prevalence of DV supports the need to identify the determinants of these behaviors to guide the implementation of efficient preventive strategies. The current study specifically explored the effect of neighborhood social support and social participation on different forms of DV perpetration and victimization, factors that have been understudied to date. Our results revealed significant associations with DV perpetration but not victimization. We also observed that these associations are gender-sensitive and depend on the form of DV considered.

High neighborhood social support was associated with a lower risk of perpetrating psychological DV among girls. To date, there are few studies on the relationship between social support and DV, and most of them focused on the effect of support from peers or family, suggesting mainly the effects of peer influence and parental supervision (Hébert et al., 2019; Richards & Branch, 2012; Richards et al., 2014). The only study on the relationship between neighborhood social support and DV that we identified (Banyard et al., 2006) failed to find a significant association. However, only instances of physical and sexual DV were assessed in this study, whereas our results suggest an association between neighborhood social support and psychological DV only. Similarly, a protective effect of perceived neighborhood social support on psychological but not on physical forms of intimate partner violence has been observed in adult samples (Kirst et al., 2015). Finally, we found that neighborhood social support appears to serve a function for adolescent girls but not boys. Richards and Branch (2012) also observed this gender sensitivity when exploring the role of peer social support. They suggested that this difference may be explained by a different perception of social support among girls and boys and by the fact that girls tend to seek more social support than boys. A similar mechanism could occur with adults in neighborhoods who could serve as role models for nonviolent conflict management and resolution. A strong presence of positive role models in neighborhoods could therefore promote a reduction in the use of psychological DV in the context of romantic relationships. Thus, our findings suggest that social networks outside the family and peers, particularly adults in neighborhoods, may influence the prevalence of DV and could complement interventions targeting peers and family. However, the effect of neighborhood social support was not observed for physical/sexual DV perpetration nor for DV victimization. Social support from peers and family, for which effects have been observed in some studies (Hébert et al., 2019; Richards & Branch, 2012; Richards et al., 2014), may further affect these forms of DV. Also, the entire social support network may play a role on experiences of violence in romantic relationships, and a greater number of network members could help to disclose and reduce adverse outcomes related to these experiences (Sylaska & Edwards, 2014). Adults in neighborhoods are part of these networks, and our results highlight their important role in preventing DV.

Associations between social participation and DV perpetration have been observed, but the direction of the relationship and the type of DV involved varied by gender. Among girls, high social participation was linked to a decreased risk of perpetrating physical/sexual DV. Conversely, for boys, high social participation was associated with a heightened risk of perpetrating psychological DV. These results highlight the importance of analyzing the association between social participation and DV by considering the modifying effect of gender. In effect, unstratified models using gender as an adjustment variable would have masked the effect of this factor (unstratified analyses in this study led to nonsignificant results). This gender sensitivity could be explained by the norms specific to the environments frequented by adolescents. Gender norms and norms supportive of violence, for example, may be more prevalent in male peer groups such as some sports clubs (De Keseredy & Kelly, 1995). Social participation could contribute to boys’ exposure to these norms, which act as a risk factor for DV. Conversely, girls may be more likely to benefit from the protective effects of social participation. Social participation would promote supervision of adolescents and foster the development of social ties (Browning & Soller, 2014; Quane & Rankin, 2006). These social ties may encourage prosocial behaviors that may help reduce girls’ risk of perpetrating DV. Finally, our results did not find associations between social participation and DV victimization. However, studies found that specific sports environments may have a protective effect on victimization among girls (Milner & Baker, 2015). The absence of such associations in our study could be explained by the measure of social participation, which was limited in identifying the effect of specific environments.

### Strengths and Limitations

The influence of neighborhood social support and social participation on DV have been poorly documented in the scientific literature, and our analyses demonstrated their potential role in this phenomenon, thus offering new avenues for intervention. This study also analyzed the effect of social support and social participation by distinguishing several forms of DV, and the results suggested that psychological DV may be more sensitive to these factors. In addition, the effect sizes were considerable, suggesting that these factors may have a critical influence, especially for girls. These findings are interesting given that psychological DV is the most prevalent form of DV and is often seen as a precursor to other forms of DV (O’Leary & Smith Slep, 2003), and preventive strategies that help reduce the occurrence of psychological DV may help to limit the development of more severe forms. However, this form of DV has rarely been analyzed independently in studies of the relationship between neighborhoods and DV (Johnson et al., 2015). Therefore, future studies should assess the influence of socioenvironmental factors on each form of DV separately to more adequately describe these relationships.

Despite these strengths, this study has some limitations. One limitation is the cross-sectional nature of the data which limits causal inference as some aspects of DV (e.g., controlling behavior) can lead to social isolation (Saltzman et al., 2002). Experiencing DV may limit adolescents’ ability to seek and obtain social support and their ability to engage in activities, thereby reducing their social participation. Future investigations using longitudinal data are required for clarifying the causal nature of these relationships.

Another limitation is the relatively small sample size, which resulted in a decrease in statistical power and an increase in the risk of Type II error. Due to the sample size, it was also not possible to analyze physical and sexual DV separately. Knowledge about the specific factors associated with each of these forms of violence is still limited. However, neighborhood social support and social participation may differently influence physical and sexual DV. Therefore, future research should assess the effect of these factors by distinguishing the different forms of violence.

Another limitation relates to the measures used to operationalize social support and social participation. This information was provided by adolescents themselves and was limited in describing neighborhood-level processes as it reflects their own perceptions and behaviors. Aggregating individual data to the neighborhood level (e.g., average score for each neighborhood) would have addressed this issue by assessing these social processes at the neighborhood level (Diez Roux, 2007), but the density of participants was not sufficient for doing so. The development of such measures should be a priority to describe socioenvironmental processes and their effect on DV. In addition, the measure of social participation did not distinguish between the types of environments in which adolescents were involved (e.g., sports, religion, arts). However, our results suggested opposite effects for boys and girls, which could be explained by the characteristics of these different contexts. Future studies should examine the effect of each context with the assistance of more comprehensive measures to identify the most vulnerable environments and for informing targeted interventions.

### Implications for Research and Practice

Neighborhood social support and social participation are determinants of DV perpetration. Exploring the mechanisms by which these factors influence DV is an avenue of research that may lead to a better understanding of the effects of the social environment and help prevent these behaviors. Specifically, neighborhood social support could reduce DV by increasing adolescents’ supervision and help-seeking (Browning, 2002; Wright et al., 2015). Regarding social participation, the characteristics of context in which adolescents engage in activities could play an important role in the relationship between social participation and DV. To test these potential mechanisms, path analyses should be implemented in the future studies. In addition, the effect of social support should be further investigated by considering the density and the diversity of adolescents’ social network (e.g., neighbors, family, peers). Such analyses would provide a better understanding of the influence of social support on DV, and a more nuanced description of this relationship. Finally, social support and social participation are not the only dimensions of the social environment. At the neighborhood level, Carpiano (2006) identified two additional components: informal social control, which refers to a community’s ability to maintain social order collectively, and social leverage, which relates to residents’ ability to access socioeconomic benefits or information. Assessing the effect of these components on DV as well as the combined effect of different forms of the social environment may contribute to further understand the role of the neighborhoods’ social environment in the occurrence of DV.

The results of our study also have implications for intervention. Findings highlight the importance of improving social support and social participation at the neighborhood level to prevent DV among girls. Mentoring programs implemented in some communities (e.g., Big Brothers Big Sisters) (Schwartz et al., 2012) could provide social support and guidance to the most vulnerable adolescents. Moreover, the development of community organizations, clubs, and structured activities in neighborhoods could promote social participation and social interaction among adolescents (Browning & Soller, 2014; Quane & Rankin, 2006). These environments could also be used to encourage respectful, non-abusive, and egalitarian relationships and implement DV prevention. For boys, efforts should mainly target at-risk male peer groups, in which adolescents are more likely to adhere to gender norms and acceptance of violence. Programs offered to high school athletes to increase knowledge about DV and promote positive behaviors among witnesses to violence have offered promising approaches to preventing the perpetration of DV (Jaime et al., 2018). Such interventions could be implemented in out-of-school clubs, sports, and community organizations and thus target other adolescents. These preventive measures could complement existing intervention programs by targeting different environments in which adolescents live. Such endeavors may ultimately contribute to eradicate violence in the romantic relationships of teenagers.
